# Mesenchymal Stem Cells Augment the Anti-Bacterial Activity of Neutrophil Granulocytes

**DOI:** 10.1371/journal.pone.0106903

**Published:** 2014-09-19

**Authors:** Sven Brandau, Mark Jakob, Kirsten Bruderek, Friedrich Bootz, Bernd Giebel, Stefan Radtke, Katharina Mauel, Marcus Jäger, Stefanie B. Flohé, Stephan Lang

**Affiliations:** 1 Department of Otorhinolaryngology, University Hospital Essen, Essen, Germany; 2 Department of Otorhinolaryngology, University Hospital Bonn, Bonn, Germany; 3 Institute of Transfusion Medicine, University Hospital Essen, Essen, Germany; 4 Surgical Research, Department of Trauma Surgery, University Hospital Essen, Essen, Germany; Hannover Medical University (MHH), Germany

## Abstract

**Background:**

Mesenchymal stem cells (MSCs) participate in the regulation of inflammation and innate immunity, for example by responding to pathogen-derived signals and by regulating the function of innate immune cells. MSCs from the bone-marrow and peripheral tissues share common basic cell-biological functions. However, it is unknown whether these MSCs exhibit different responses to microbial challenge and whether this response subsequently modulates the regulation of inflammatory cells by MSCs.

**Methodology/Principal Findings:**

We isolated MSCs from human bone-marrow (bmMSCs) and human salivary gland (pgMSCs). Expression levels of TLR4 and LPS-responsive molecules were determined by flow cytometry and quantitative PCR. Cytokine release was determined by ELISA. The effect of supernatants from unstimulated and LPS-stimulated MSCs on recruitment, cytokine secretion, bacterial clearance and oxidative burst of polymorphonuclear neutrophil granulocytes (PMN) was tested *in vitro*. Despite minor quantitative differences, bmMSCs and pgMSCs showed a similar cell biological response to bacterial endotoxin. Both types of MSCs augmented anti-microbial functions of PMNs LPS stimulation, particularly of bmMSCs, further augmented MSC-mediated activation of PMN.

**Conclusions/Significance:**

This study suggests that MSCs may contribute to the resolution of infection and inflammation by promoting the anti-microbial activity of PMNs. This property is exerted by MSCs derived from both the bone-marrow and peripheral glandular tissue.

## Introduction

Mesenchymal stem cells (MSCs) are multipotent, non-hematopoietic, plastic adherent fibroblast-like progenitor cells capable of differentiation into mesenchymal and non-mesenchymal lineages [1], [2]. At present, no unique marker exists to clearly separate MSCs from other cell types. MSCs are instead defined by specific combinations of morphologic, immunophenotypic, and functional properties [3]. They are characterized by the absence of specific endothelial and hematopoietic cell surface antigens (CD11, CD14, CD31, CD34, and CD45) and the expression of certain cell surface markers (CD29, CD44, CD105, CD73, CD90, and CD106) [4]. They can differentiate into many cell lineages, including chondrocytes, osteoblasts, adipocytes, tenocytes, and myocytes, when seeded in the appropriate differentiation media [2].

Based on their proposed broad, multi-lineage differentiation potential MSCs have become one of the most intensively studied adult stem cell entity throughout the last 15 years [5]. Based on the assumption that MSCs contain the potential to replace lost cells in various tissues, they are administered in a variety of clinical settings. Remarkable successes have already been achieved by the systemic or local application of MSCs in cases of graft-versus-host disease (GvHD) [6], Crohn's disease [7] and chronic wounds [8], such as diabetic ulcers [9]. However, administered MSCs get rapidly trapped in the lung and are rarely recovered in damaged target tissues. This somewhat contradicts the initial hypothesis that MSCs replace lost cell types. Alternatively, it has been proposed that MSCs improve clinical outcomes by paracrine effects rather than by their previously proposed engraftment into damaged host tissues. Indeed, it is now widely assumed that MSCs secrete a number of soluble factors, which regulate inflammation and immune responses [5], [10], .

In addition to this, MSCs display immunomodulatory properties through direct interaction with a variety of leukocyte subsets [12]. MSCs have mostly been defined as immunosuppressive, as they down-regulated functions of dendritic cells [13], T cells [14], NK cells [15], and B cells [16]. In addition to mononuclear cells, MSCs may also interact with polymorphonuclear neutrophil granulocytes (PMNs), which play an important role in the acute inflammatory response and in the clearance of bacterial infections. Indeed, recent studies have shown the modulation of PMN functions by MSCs [17]–[20].

MSCs reside mainly in the bone marrow (bmMSCs) but are able to enter inflamed, traumatized or malignant target tissues through the circulation. In addition to bone marrow, MSCs have been isolated from many peripheral tissues such as salivary glands [21], nasal mucosa [22], adipose tissue [23], liver, spleen [24], placenta [25], umbilical cord blood [23], and peripheral blood [26]. While the immunomodulatory function of bmMSCs is well characterized, the role and function of these tissue-specific MSCs still remains elusive.

In the present study, we sought to investigate the similarities and differences between bmMSCs and tissue-resident MSCs from the parotid gland (pgMSCs), as well as their response to bacterial lipopolysaccharide (LPS) as a model system for bacterial infection and inflammation. In the second part of the study we analysed the consequences of LPS stimulation on the interaction of MSCs and PMNs. To this end we set up an in vitro system of LPS stimulation and MSC-PMN interaction.

Our results show that both MSC types display a comparable response to LPS and enhance the anti-bacterial activity of PMNs. These findings open the possibility that a MSC-PMN interplay may contribute to the clearance of bacterial infection.

## Results

### Characterization of bmMSCs and pgMSCs

**Figure 1:**
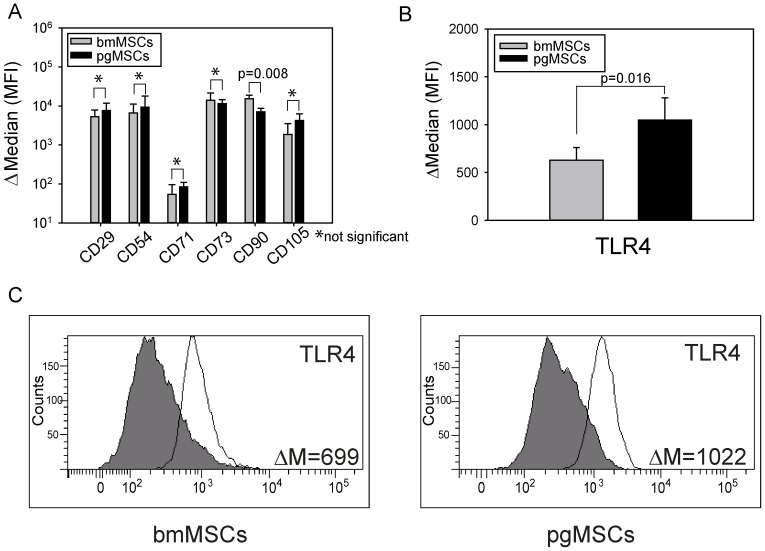
Immunophenotyping of bmMSCs and pgMSCs. (A/B) Flow cytometric analysis of MSCs; data are presented as Δ Median defined as the mean fluorescence intensity (MFI) of the specific antibody minus MFI of the isotype control antibody. Bar graphs show the median from 4–5 independent experiments (bmMSCs, n = 4; pgMSCs, n = 5). (B/C) TLR4 expression of bmMSCs and pgMSCs was determined by flow cytometry (five independent experiments). The Mann-Whitney U test was used for statistical analysis; significance was set at the level of *P*≤0.05. (C) Data are shown as an overlay histogram: isotype control (gray) and specific cell surface marker for TLR4 (white). Representative result from 1 out of 5 independent experiments (bmMSCs, n = 5; pgMSCs, n = 5).

Flow cytometry of MSCs (bmMSCs n = 4; pgMSCs n = 5; passage 3–5) demonstrated that bmMSCs and pgMSCs expressed typical MSC marker proteins (Figure 1 A). BmMSCs showed a significantly higher constitutive expression of CD90 (Thy-1) (p = 0.008; Mann-Whitney U test), whereas CD29, CD54, CD71, CD73, and CD105 expression levels were similar between MSC subtypes. Markers for endothelial cells (CD31), hematopoietic cells (CD45), and hematopoietic stem cells (CD34) were absent (data not shown), whereas both bmMSCs and pgMSCs were positive for CD29, CD71, CD73 and CD90 (Figure 1 A).

### TLR4 expression of human bmMSCs and pgMSCs

As we intended to use LPS as a bacterial model stimulus, we first examined the expression of its receptor, TLR4, by flow cytometry (Figure 1 B/C). Our data showed that TLR4 was strongly expressed by both MSC populations. However, we detected a significantly higher TLR4 expression in pgMSCs (p = 0.016).

### Expression of adhesion molecules

**Figure 2:**
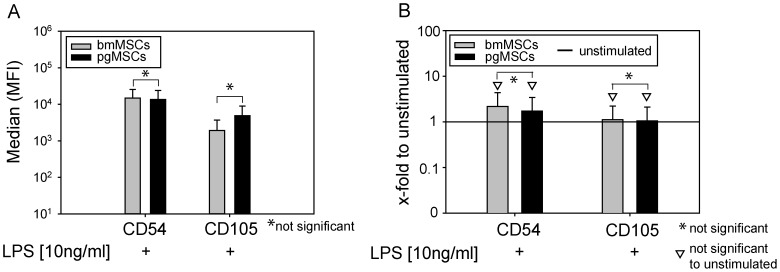
Expression of adhesion molecules after LPS stimulation. MSCs were left untreated or were stimulated with LPS [10 ng/ml]. Flow cytometry data are presented as ΔMedian of 5–7 independent experiments (bmMSCs, n = 5; pgMSCs, n = 7). (A) Absolute fluorescence intensity is shown. (B) Relative induction of fluorescence intensity with untreated MSCs set as 1 (reference value) is shown for visualization of LPS-induced up-regulation; Mann-Whitney U test was used for statistical analysis; significance was set at the level of *P*≤0.05.

MSCs are known to express a set of receptors associated with matrix- and cell-to-cell adhesive interaction. These receptors are frequently up-regulated during cellular activation with LPS and thus may serve as surrogate markers for an activated state. In order to test a possible differential constitutive activation of MSCs isolated from different source tissues we compared the adhesion molecules CD50 (ICAM-3), CD54 (ICAM-1), CD56 (NCAM), CD62L (L-Selectin), and CD105 (SH2; endoglin). Both, bmMSCs and pgMSCs, constitutively expressed the adhesion molecules CD54 (ICAM-1) and CD105 (SH2; endoglin) at similar levels (Figure 1 A), while they were negative for CD50 (ICAM3), CD56 (NCAM) and CD62L (L-Selectin) (data not shown). Next, we tested the expression of CD54 and CD105 on bmMSCs and pgMSCs after exposure to LPS (10 ng/ml for 24 hours). Figure 2 A shows the expression of CD54 and CD105 in bmMSCs and pgMSCs after LPS as Δ Median (MFI). There were no significant differences in expression levels between pgMSCs and bmMSCs (Figure 2 A). There was a trend towards higher expression of CD54 after LPS stimulation, which did not reach statistical significance (bmMSCs: p =  0.055 and pgMSCs: p =  0.068; Mann-Whitney U test). CD105 expression remained unaltered (bmMSCs: p =  0.397 and pgMSCs: p =  0.473; Mann-Whitney U test) (Figure 2 B).

### Induction of cytokine release and activation of signal transduction pathways by LPS

**Figure 3:**
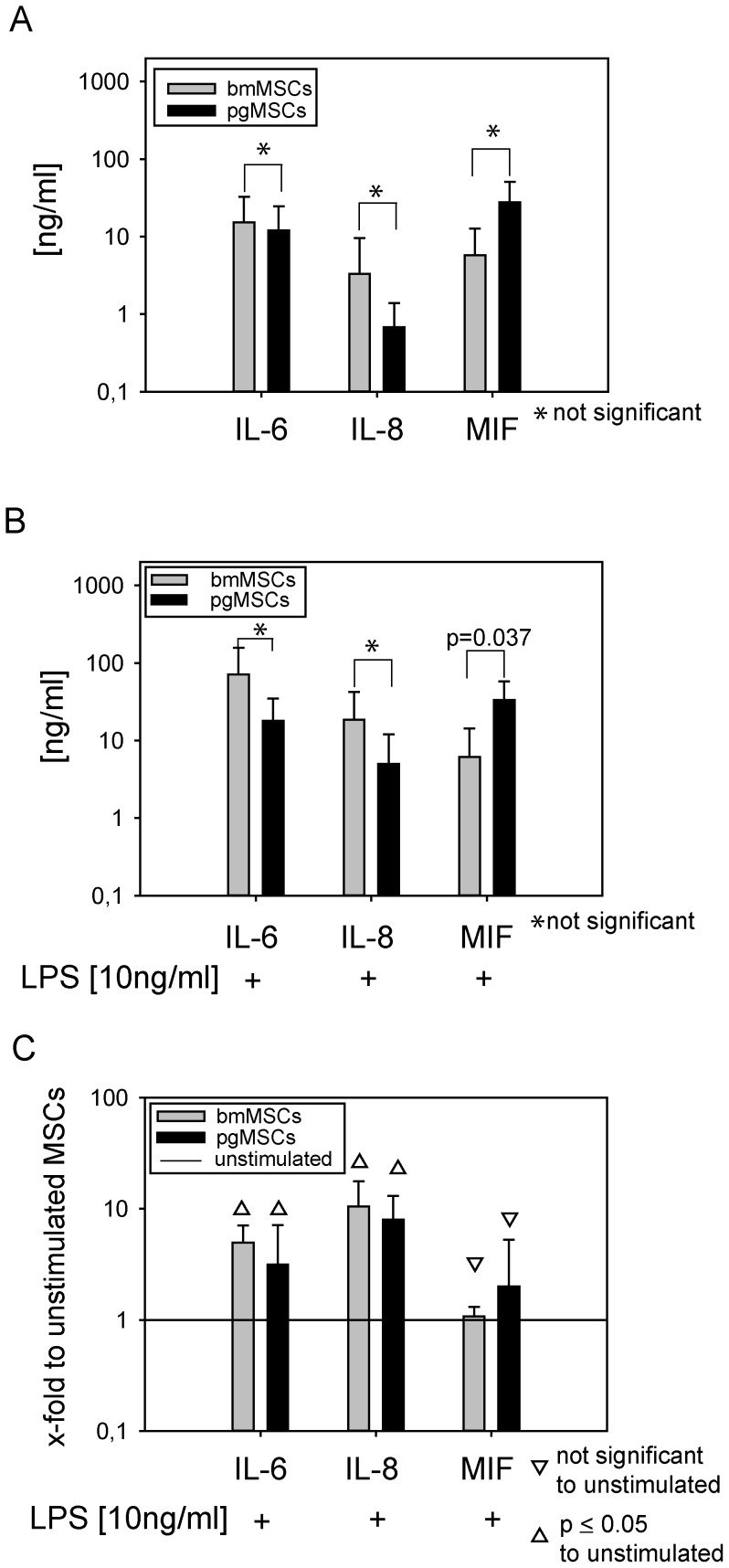
Cytokine secretion profile of bmMSCs and pgMSCs. Cytokines were quantified by ELISA. MSCs were (A) left untreated or (B/C) were stimulated with LPS [10 ng/ml] (bmMSCs, n = 7; pgMSCs, n = 5; passage 3). (C) Cytokine secretion of untreated MSCs was set as 1 (reference value). Mann-Whitney test was used for statistical analysis; significance was set at the level of *P*≤0.05.

Next we investigated inflammatory cytokine release and TLR4 downstream signaling pathways in bmMSCs and pgMSCs. High levels of IL-6, IL-8 and MIF were produced, even in the absence of stimulation (Figure 3 A). After exposure to the endotoxin LPS, secretion of IL-6 and IL-8 was significantly induced in bmMSCs and pgMSCs (Figure 3 B/C). Interestingly, pgMSCs secreted higher amounts of MIF than bmMSCs did after LPS stimulation (p = 0.037).

**Figure 4:**
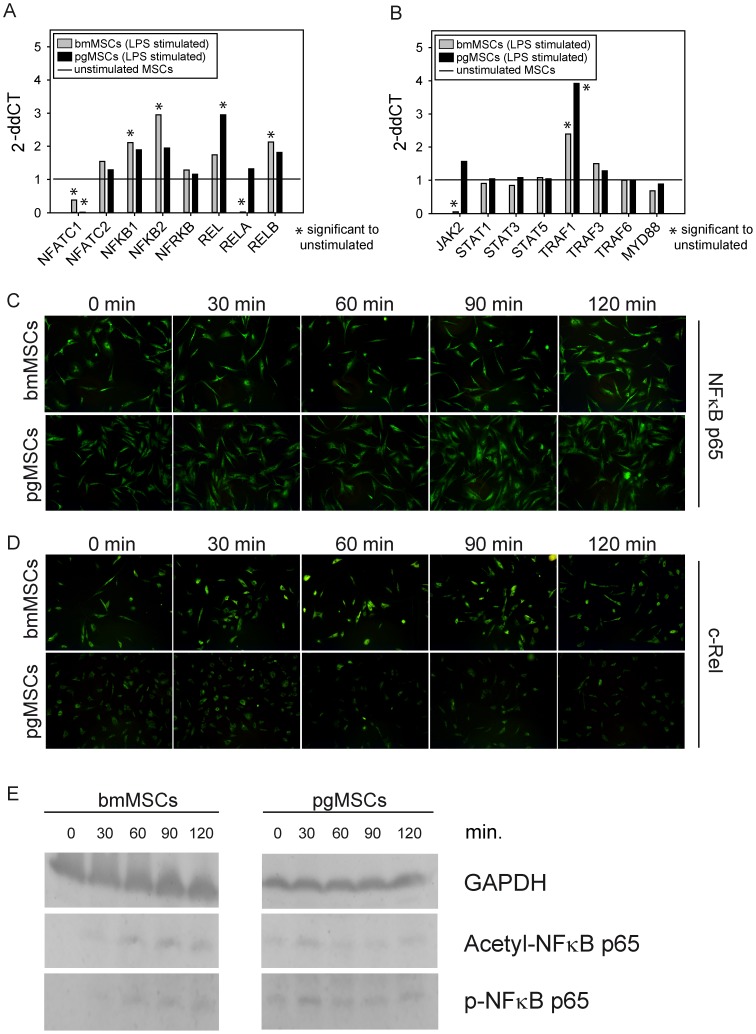
Activation of the NF-κB, JAK-STAT and TRAF pathways. (A/B) mRNA expression of bmMSCs and pgMSCs was determined in unstimulated and stimulated (LPS, 10 ng/mL for 4 h) MSCs by qPCR. mRNA was pooled from 4 different donors respectively for bmMSCs and pgMSCs. Gene expression of unstimulated MSCs was set as 1 (reference value). Values ≥ 2.0 are set as a significant increase of gene expression and values≤0.5 are set as a significant decrease of gene expression after LPS stimulation. 2^−ddCT^ = 2^−(dCT Target – dCT Reference) LPS stimulated – (dCT Target – dCT Reference)^ ^unstimulated^. (C/D) Classical (canonical) NF-κB pathway: immunofluorescence analysis of translocation of p65 (C) and c-REL (D) was determined in unstimulated and stimulated (LPS, 10 ng/mL for 0, 30, 60, 90, 120 min) bmMSCs and pgMSCs. One representative experiment of two independent experiments is shown. (E) Analysis of the phosphorylation status of Acetyl-NF-κB p65 and phospho-NF-κB (p-NF-κB) detected by western blot (unstimulated and stimulated with LPS for 0, 30, 60, 90, and 120 min).

Subsequently, the expression of components from the NF-κB, JAK-STAT and TRAF pathways of bmMSCs and pgMSCs after exposure to LPS were investigated by qPCR (Table 1). As shown in figure 4 a-b, LPS challenge modulates the mRNA expression levels of several signalling molecules. Specifically we observed up-regulation of NFKB1, NFKB2, REL, RELB and TRAF1. However, the exposure of MSCs to LPS did not affect the expression levels of mRNA of the JAK-STAT signaling pathway. BmMSC expression levels of mRNA of NFKB1, NFKB2 and RELB were significantly higher compared to pgMSCs. Next we analyzed nuclear translocation of NF-κB p65 and c-REL by immunofluorescence (Figure 4 C/D). Unexpectedly, we found only minimal cytoplasmic-to-nuclear translocation of both molecules in bmMSCs as well as pgMSCs. These findings were confirmed when we observed only slight acetylation of NF-κB p65 and phosphorylation of the p65 subunit (figure 4 E) after stimulation with LPS. Collectively these data suggest that substantial LPS-induced cytokine release in MSCs is accompanied by only moderate molecular activation of the classical NF-κB pathway.

**Table 1:** Primerlist.

	Primer sequence			
Target	Sense	Antisense		Tm°
NFATC1	CACCAAAGTCCTGGAGATCCCA	TTCTTCCTCCCGATGTCCGTCT		60
NFATC2	GATAGTGGGCAACACCAAAGTCC	TCTCGCCTTTCCGCAGCTCAAT		60
NFKB1	GCAGCACTACTTCTTGACCACC	TCTGCTCCTGAGCATTGACGTC		60
NKFB2	GGCAGACCAGTGTCATTGAGCA	CAGCAGAAAGCTCACCACACTC		60
NFRKB	CTCCACCTGTATCGGCAGTGAA	GTTCCCAGATGTGGCATTGTTGG		60
REL	CGAACCCAATTTATGACAACCG	TTTTGTTTCTTTGCTTTATTGCCG		60
RELA	TGAACCGAAACTCTGGCAGCTG	CATCAGCTTGCGAAAAGGAGCC		60
RELB	TGTGGTGAGGATCTGCTTCCAG	TCGGCAAATCCGCAGCTCTGAT		60
JAK2	CCAGATGGAAACTGTTCGCTCAG	GAGGTTGGTACATCAGAAACACC		60
STAT1	TCTCGGATAGTGGGCTCTGT	CTATCAACAGGTTGCAGCGA		63
STAT3	CCCCCGCACTTTAGATTCAT	GGTAGGCGCCTCAGTCGTAT		63
STAT5	GCCACTGTTCTCTGGGACAATG	ACACGAGGTTCTCCTTGGTCAG		60
TRAF1	CGATGGCACTTTCCTGTGGAAG	TACAGCCGCAGGCACAACTTGT		60
TRAF3	ACAAGTGCAGCGTCCAGACTCT	CAATGCCAGCGTCCCTTCCAAA		60
TRAF6	CAATGCCAGCGTCCCTTCCAAA	CCAAAGGACAGTTCTGGTCATGG		60
MYD88	CGCCGGATGGTGGTGGTTGT	TGTAGTCGCAGACAGTGATGAACC		60
Beta 2M	AGCGTACTCCA AGATTCAGGTT	ATGATGCTGCTTACATGTCTCGAT		60/63
B-Actin	AGCGGGAAATCGTGCGTG	GGGTACATGGTGGTGCCG		60

Tm° indicates melting temperature in degrees Celsius.

### Modulation of anti-microbial PMNs activity by MSCs

PMNs are central cellular effectors of infection and inflammation. Therefore, in the final part of our study, supernatants from LPS challenged MSC were used to assess the modulation of these bona fide inflammatory cells by MSCs.

**Figure 5:**
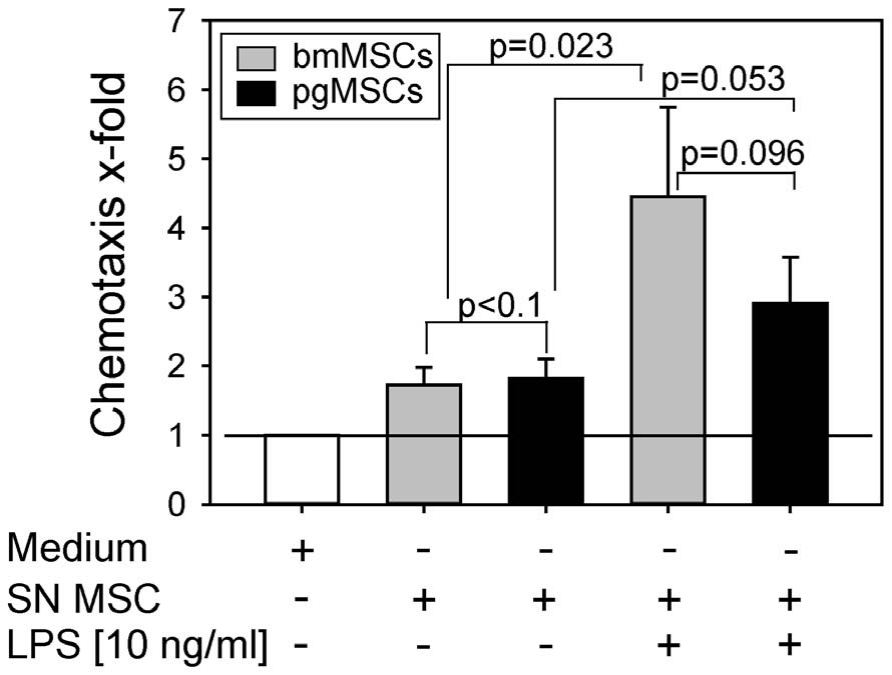
Activated bmMSCs and pgMSCs induced the recruitment of PMNs. A migration assay using a Transwell insert culture system was used to evaluate the migration of PMNs toward medium, MSCs (bmMSCs and pgMSCs, each n = 5) supernatant (SN), and supernatant of LPS-stimulated MSCs. Data are presented as median of 5 independent experiments. Spontaneous migration toward control medium was set as 1 (reference value); statistical analysis by ANOVA test; significance was set at the level of *P*≤0.05.

Using a Transwel insert culture system, we observed that the supernatant of unstimulated bmMSCs and pgMSCs induced moderate chemotaxis of PMNs (Figure 5). No difference between bmMSCs and pgMSCs was detected. Notably, the supernatant obtained from both MSC populations after stimulation with LPS clearly enhanced the recruitment and directed migration of PMNs (p =  0.023 for bmMSCs; p =  0.053 for pgMSCs). The supernatant of LPS-stimulated bmMSCs induced stronger PMN chemotaxis compared to supernatant of LPS-stimulated pgMSCs.

**Figure 6:**
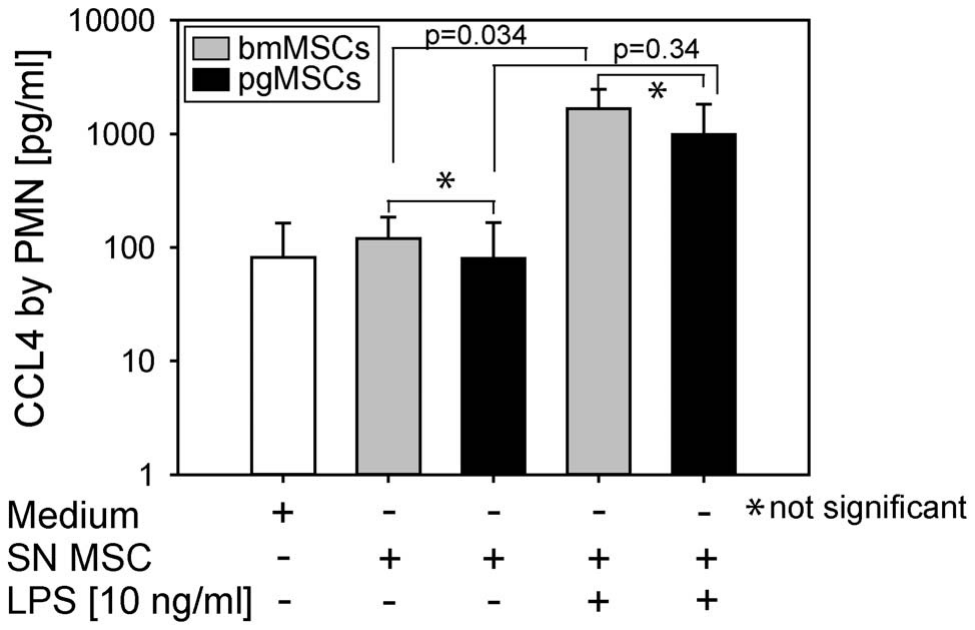
CCL4 secretion by PMNs. PMNs were cultured in control medium or in conditioned medium of unstimulated or LPS-stimulated MSCs for 24 hours. CCL4 secreted by PMNs was measured by ELISA. Data are presented as median of 3 independent experiments. ANOVA test was used for statistical analysis; significance was set at the level of *P*≤0.05.

Next, we asked whether stimulated MSCs also modulate the inflammatory and anti-microbial activity of PMNs. CCL4 (MIP-1β) is a proinflammatory chemokine that binds to CCR5 and mediates the recruitment of macrophages, DCs, and activated T-cells. Unstimulated PMNs produced only small amounts of CCL4. CCL4 production was not upregulated when PMNs were exposed to a culture medium conditioned by unstimulated bmMSCs or pgMSCs. However, if PMNs were exposed to the supernatant of bmMSCs or pgMSCs, previously stimulated with 10 ng/mL LPS, CCL4 secretion by PMNs was strongly induced (Figure 6). However, LPS-mediated paracrine induction of CCL4 in PMNs only reached statistical significance for bmMSCs and not for pgMSCs.

**Figure 7:**
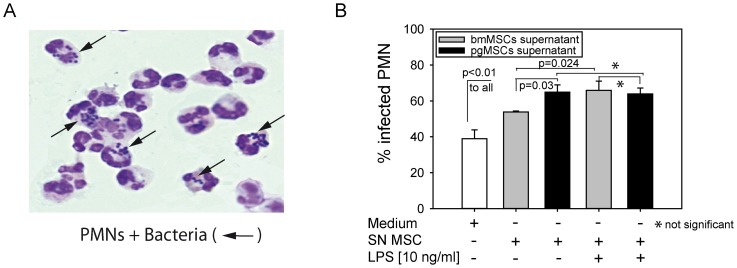
Phagocytic activity of PMNs. (A) PMNs were pre-incubated in MSCs (bmMSCs or pgMSCs) supernatant or standard culture medium (1 h). Then PMNs were infected with Escherichia coli (MOI 50) for 30 minutes. Phagocytosis was assessed and counted after Pappenheim's staining. Examples of Pappenheim's-stained PMNs (arrow: phagocytized bacteria). One representative photomicrograph from 3 different donor cultures is shown (63× magnification). MOI, multiplicities of infection. (B) Influence of bmMSC or pgMSC supernatants (SN) on the phagocytic activity of PMNs. Data are depicted as percentage of infected PMNs (n = 3).

To evaluate whether supernatant of unstimulated and LPS-stimulated MSCs influences the phagocytic function of PMNs, PMNs were isolated and incubated for 1 h in MSCs supernatant or unconditioned medium, and subsequently incubated with bacteria for 30 min. The efficacy of phagocytosis was assessed by counting intracellular bacteria taken up by PMNs (Figure 7 A). Under unstimulated control conditions less than 40% PMNs contained intracellular bacteria. In the presence of MSCs-conditioned medium this number increased to more than 60% for pgMSCs suggesting a profound activation of anti-microbial activity in PMNs. In bmMSCs LPS-stimulation resulted in a further increase of PMN phagocytosis (p = 0.024) (Figure 7 B).

**Figure 8:**
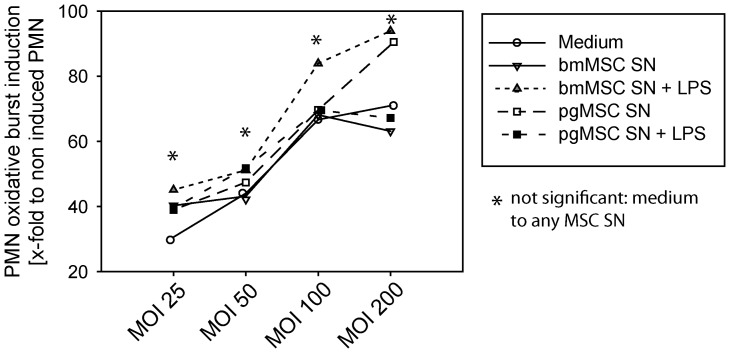
Respiratory Burst. PMNs were pre-incubated in bmMSCs or pgMSCs supernatant or standard culture medium (1 h). PMNs were incubated in the presence or absence of Escherischia coli with MOI 25, 50, 100, and 200, followed by addition of dihydrorhodamine-123. Superoxide anion (O_2_^−^) release was measured by flow cytometry. Each panel of the figure shows the mean of 3 independent experiments.

To test and induce a respiratory burst in PMNs, a bacterial suspension was added at multiplicities of infection (MOIs) ranging from 25∶1 to 200∶1. Under these conditions, bacterially challenged PMNs showed the strongest respiratory burst in medium conditioned by LPS-stimulated bmMSCs (Figure 8). Collectively, this final series of experiments suggests LPS stimulation augments the capacity of MSCs to activate inflammatory and anti-microbial PMN effector functions. This effect is more pronounced for bmMSCs as compared to pgMSCs.

## Discussion

In recent years, MSCs have been isolated, expanded and characterized from various peripheral tissues besides the well-characterized bone marrow MSCs (bmMSCs) [22], [23], [30]. Their immunomodulatory potential, together with their ease of isolation and expansion, make MSCs an attractive tool for the treatment of inflammatory and autoimmune diseases [6], [7], [31]. Not surprisingly, cellular therapy with MSCs is currently being evaluated in numerous clinical and preclinical studies. These studies established that adoptively transferred MSCs can down-regulate unwanted immune activity [7], [32].

MSCs exert immunoregulatory effects through direct and indirect interaction with other immune cells. Furthermore, MSCs seem to play an important role in wound healing, tissue regeneration, resolution of inflammation and the clearance of bacterial infections [33], [34]. Systemically applied MSCs accumulate at sites of tissue damage or infection in order to exert their anti-inflammatory, immunoregulatory, and regenerative effects [35], [36].

In our study, we were interested in analyzing the functional and cell biological differences and similarities between tissue resident MSCs (pgMSCs) and bmMSCs. Most surface molecules and cytokines were similarly expressed between both MSC types. Interestingly, pgMSCs showed higher surface expression of TLR4, the receptor for LPS. However, in stimulation experiments, we found that MSCs from both body compartments readily responded to bacterial endotoxin. Unexpectedly, our preliminary data on LPS-induced signalling indicated only a moderate activation of the classical canonical NFκB pathway [45] in both MSC types. In addition to the classical pathway, activation of human MSCs by LPS may also occur via the noncanonical pathway [46]. While our RNA data indicate expression of several members of this signalling pathway in our MSCs, further studies are needed to delineate the exact molecular events operative in human MSCs after challenge with LPS.

In previous studies we found that stimulation of pgMSCs with LPS increased their capacity to recruit and sensitize PMNs for further LPS challenge [17]. We have now demonstrated that neutrophil activation is shared by bmMSCs. Our data indicate that not only are the molecular and immunological responses of pgMSCs and bmMSCs to LPS similar, but, most importantly both types of MSCs enhance the anti-microbial activity of PMNs. Interestingly, there was a trend towards greater LPS-mediated activation of PMN effector function for bmMSCs compared with pgMSCs. These findings are not necessarily expected, as we originally speculated that MSCs isolated from peripheral tissues are more likely to be exposed to external pathogens and thus might display a stronger response to pathogenic structures. However, our data suggest that the capacity of MSCs to augment inflammatory and anti-microbial functions of PMNs is not restricted to MSCs of peripheral tissues, but is also an inherent feature of bmMSCs. It is important to note that the data compared between pgMSCs and bmMSCs applies the culture and experimental conditions used in our study. Culture conditions affect cell biological functions of MSCs: this has been most extensively studied for culture media supplements such as platelet lysate or the replacement of serum containing media by serum-free media [27]–[29]. Comparative studies are complicated by the fact that many different culture conditions have been report for MSCs and MSCs obtained from diverse tissue sources are often cultured under different conditions. For this reason, we decided to use simple, standard culture conditions with pre-tested identical FCS charges for both types of MSCs even though more sophisticated culture conditions have been reported for bmMSCs, which would have allowed for more efficient expansion [27]–[29].

A major finding of this study is the capacity of MSCs to enhance bacterial uptake by PMNs (Figure 7). In addition, MSCs secrete large amounts of IL-6, IL-8 and MIF cytokines, which are implicated in the recruitment and activation of PMNs [10], [17], [19]. IL-8 is a potent chemoattractant for PMNs and promotes PMNs recruitment by binding to the receptors CXCR1 and CXCR2. Recruitment of PMNs is also triggered by other chemoattractant mediators like chemokines, lipids or complement anaphylotoxins [37], [38]. Our data suggest that, in addition to IL-8, other chemokines and CXCR1/2 ligands are contributing to the PMNs recruitment. Both MSCs types secrete MIF (macrophage migration inhibitory factor). MIF was originally identified as an inhibitor of random macrophage migration *in vitro* and is best known for its role in microbial sepsis. However, newer studies revealed that MIF is a ligand for CXCR2 – one of the major chemokine receptors expressed on PMNs – and, thus, is able to modulate PMNs chemotaxis [39], [40].

In conjunction, these findings suggest that MSC may engage in a cross-talk with phagocytic granulocytes, resulting in enhanced clearance of bacteria. Altogether, these recent findings suggest the attractive hypothesis that tissue resident MSCs act as local regulators of immunity in infected tissues. According to this hypothesis, the sessile MSCs would be exposed to incoming pathogens and, after appropriate activation, direct the influx and modulate the activity of motile incoming immune cells with implications for anti-bacterial immunity and the regulation of associated inflammatory processes. This more recently emerging inherent physiological function of MSCs would complement two well-recognized and previously reported functions of MSCs: support of hematopoiesis for bmMSCs [41] and down-regulation of unwanted immunity by MSCs from various sources applied as cellular therapeutics in hyper-immune pathologies such as GvHD [6], autoimmunity or even sepsis [42].

## Material and Methods

### MSCs preparation and culture conditions

Bone marrow was harvested from iliac crest of healthy donors (n = 7) at the Department of Orthopaedics, University Hospital Essen, Germany. Mononuclear cells were isolated by density gradient centrifugation (Ficoll-Paque, GE-Healthcare, Munich, Germany). Cells were seeded in standard culture medium (low-glucose DMEM (Invitrogen, Karlsruhe, Germany) supplemented with 10% foetal bovine serum (FBS; Biochrom, Berlin, Germany), 1% Penicillin/Streptomycin (Invitrogen), 1% L-glutamine (Invitrogen)). During the culture period, cells were maintained at 37°C in a humidified atmosphere of 5% CO_2_. After 72 hours, non-adherent cells were removed by washing, and medium was changed twice a week. BmMSCs were continuously passaged after reaching subconfluency by Stem Pro Accutase (Invitrogen) treatment for 5 minutes at 37°C. We used protocols described previously of tissue dissociation, plastic adherence, and progenitor cell expansion to isolate and enrich MSCs from human parotid glands (n = 5) [10], [17]. Until now, no single specific marker has been identified to unequivocally distinguish MSCs from other cell types. To identify bmMSCs and pgMSCs as mesenchymal stem cells, we combined tri-lineage differentiation (osteogenic, chondrogenic, adipogenic; data not shown) with immunofluorescence (IF) and immunohistology (IHC) techniques. All donors gave a written informed consent, and the study was approved by the ethics committee of the Medical Faculty of the University Duisburg-Essen.

### Flow cytometric analysis of bmMSCs and pgMSCs

To evaluate cell-surface marker expression, bmMSCs and pgMSCs were stained with the following antibodies: CD29 (PE; β1-integrin, clone: MAR4; BD Bioscience, Heidelberg, Germany), CD31 (APC-eFlour-780; PECAM-1, clone: WM59; eBioscience, Frankfurt, Germany), CD34 (FITC; My10, clone: 581 (Class 3; Invitrogen), CD45 (V500; leukocyte common antigen, clone:HI30; BD Bioscience), CD50 (ICAM-3; PerCP-Cy5.5 clone CBR-IC3/1, BioLegend, Fell, Germany), CD54 (ICAM-1; APC clone HA58, eBioscience), CD56 (NCAM; PE clone B159, BD Bioscience, Heidelberg, Germany), CD62L (L-Selectin; V450 clone DREG-56, BD Bioscience); CD71 (APC; transferrin receptor, clone: AD2; BD Bioscience), CD73 (PerCP-eFlour-710; ecto-5-NT, SH4; clone: AD2; BD eBioscience), CD90 (Brilliant Violet 421; Thy-1, clone: 5E10; BioLegend, Fell, Germany), CD105 (PE-Cy7: Endoglin/TGF1-b3 receptor, clone: 43A3; BioLegend), and TLR4 (PE; abcam, Cambridge, UK). To determine nonspecific signals, isotype controls were used at the same concentration as the specific antibodies. Analysis was performed using a FACSCanto II flow cytometer (BD Bioscience) and Diva Software 6.0.

### LPS treatment of MSCs and generation of MSCs conditioned medium

MSC cultures were seeded into 12-well plates (Greiner Bio-One, Frickenhausen, Germany) and cultured in standard culture medium as described above. Confluent cultures were stimulated with 10 ng/mL LPS (salmonella Minnesota HL150; kindly provided by Prof. Dr. T. Gutsmann, Division of Biophysics, Research Center Borstel, Borstel, Germany) for 24 h. Supernatants were collected and stored at −20°C for analysis of cytokine release and cells were immediately analyzed by flow cytometry. To generate MSCs-conditioned medium for neutrophil functional assays and stimulation experiments, MSCs were treated with 10 ng/mL LPS for 4 h, washed extensively with PBS to remove LPS, and cultured further for 20 h before conditioned medium was collected and cleared further from debris by centrifugation. Supernatants from 7 different donors were pooled.

### Quantification of MSCs cytokines in culture supernatants

For cytokine quantification, cells were incubated for 24 hours in the presence or absence of LPS, as described above. Supernatants were collected by centrifugation, and typical inflammatory cytokines IL-6, IL-8, and MIF were quantified using ELISA kits (R&D Systems, Wiesbaden, Germany), according to the manufacturer's instructions. Mann-Whitney U test was performed for statistical analysis.

### Analysis of gene expression by quantitative RT-PCR analysis in bmMSCs and pgMSCs

To measure gene expression of immune response signaling pathway genes, we stimulated bmMSCs and pgMSCs for 6 h with LPS (10 ng/mL). For quantitative PCR analysis, total RNA was isolated from cultured cells using the RNeasy kit (Qiagen, Hilden, Germany). RNA concentration and purity were determined by measuring absorbance at 260 and 280 nm. Pooled RNA from 4 different donors was reverse-transcribed with random-hexamer primer and Superscript II RT, according to the manufacturer's instructions (Invitrogen). Quantitative real-time PCR (Light Cycler 2.0, Roche Applied Science, Mannheim, Germany) was conducted with primers specific for NF-κB, JAK-STAT and TRAF pathway genes (Table 1) and DyNamo Capillary SYBR Green Kit (Finnzymes/Thermo Scientific, Bonn, Germany) according to manufacturer's instructions. 2^−ddCT^ method was used for analysis of relative gene expression. Values ≥ 2.0 are set as a significant increase of gene expression and values ≤ 0.5 are set as a significant decrease of gene expression after LPS stimulation.

### Immunofluorescence analysis

MSCs were seeded overnight on coverslips followed by stimulation with 10 ng/mL LPS for 30-120 min. Cells were fixed and permeabilized using BD Cytofix/Cytoperm (BD Biosciences, Heidelberg, Germany). Cells were incubated with rabbit anti NF-κβ p65 (clone C-20, Santa cruz, Heidelberg, Germany) and c-Rel (Cell signaling/New England Biolabs, Frankfurt am Main, Germany) and then with FITC-labeled anti-rabbit (Dianova, Hamburg, Germany) antibodies for 1 hour and for 30 min at room temperature, respectively. Cells were mounted in Fluoprep (bioMerieux, Marcy l’Etoile, France) and analyzed by fluorescence microscopy with a Zeiss Axioscope 2 (Zeiss, Jena, Germany).

### Detection of phosphorylated proteins

For Western blot, MSCs were lysed in SDS-Lysis buffer (final concentrations, 25 mM Hepes, pH 7.3, 0.1% SDS, 1% Triton x-100, 10 mM EDTA, 10 mM Sodium – Pyrophosphate, 10 mM Sodium Fluoride, and 125 mM Sodium Chloride) containing 10% PhosSTOP (Roche Applied Science, Wiesbaden, Germany) and 1% each protease inhibitor cocktail I and III (Calbiochem/Merck Millipore Darmstadt, Germany). After boiling in SDS-sample buffer (final concentrations, 50 mM Tris, pH 6.8, 4% glycerin, 0.8% SDS, 1.6% β-ME, and 0.04% bromophenol blue) samples were analyzed by SDS-PAGE, followed by transfer to PVDF membranes (Roche Applied Science). Incubation with Acetyl-NF-κβ p65 (clone Lys310), Phospho-NF-κβ p65 (Ser536) (clone 93H1) and GAPDH (clone 14C10) (all Cell signaling) and goat anti rabbit alkaline phosphatase (Dianova) antibodies was performed for 1 h at room temperature. Chemiluminescent detection was performed with a ChemiDoc-It imaging system (UVP, LLC, Upland, CA, USA).

### PMNs isolation and culture

PMNs were enriched from citrate-supplemented peripheral blood from healthy donors by density gradient centrifugation. We used previously established protocols for isolation of PMNs [43]. Briefly, peripheral blood was diluted (1∶1 v/v) with 1× PBS, and PMNs were separated from MNCs by gradient centrifugation with Biocoll separating solution (Biochrom). Erythrocytes were sedimented with 1% polyvinyl alcohol solution (1∶1 v/v) (Sigma-Aldrich, Taufkirchen, Germany). Remaining erythrocytes were lysed with Aqua Braun (B. Braun). The resulting PMNs (purity of>98%) were cultured in RPMI-1640 (Invitrogen) supplemented with 10% FBS (Biochrom), 1% L-glutamine, and 1% penicillin-streptomycin (Invitrogen).

### PMNs chemotaxis assay

Chemotaxis of PMNs was examined by using 3-μm cell culture inserts in 24-well companion plates (both BD Bioscience) as previously described [44]. The plates were loaded with 700µL growth medium or with conditioned medium from LPS-stimulated and unstimulated bmMSCs (n = 5) or pgMSCs (n = 5). PMNs (5×10^5^ cells per 200µL) were placed in the inserts, allowed to migrate for 3 h at 37°C and migrated cells were counted (Casy Model TT; Roche Innovatis AG, Mannheim, Germany).

### Activation of PMNs by MSCs

To test whether MSCs modulate the activity of PMNs, we evaluated CCL4 secretion by PMNs, as we described previously [17]. Briefly, PMNs were cultivated in standard medium or in supernatant of untreated and LPS-treated (10 ng/mL for 24 h) MSCs (bmMSCs and pgMSCs) for 24 h. The amount of CCL4 secreted by PMNs was quantified with commercially available ELISA kits.

### Phagocytosis assay

Human PMNs (0.5×10^6^) from healthy donors were pre-incubated in MSCs (bmMSCs or pgMSCs) supernatant or standard culture medium for 1 h. After washing 3 times with 1× PBS, PMNs were allowed to adhere on cover slips in serum-free medium for 30 minutes. Then, PMNs were infected with *Escherichia coli* (strain JM109, multiplicity of infection (MOI) = 50) for 30 minutes in RPMI-1640 with 10% autologous serum. Phagocytic ability was assessed by using Pappenheim's staining to visualize intracellular bacteria. Infected and non-infected cells were counted using a Zeiss Axioscope 2 (Zeiss, Jena, Germany) using objective lenses with 63× magnification and Axio-Vision software (Zeiss).

### Measurement of respiratory burst

The release of reactive oxygen species (ROS, respiratory burst) by PMNs, induced by phagocytosis, was measured by oxidation of dihydrorhodamine-123 (DHR-123, Invitrogen) to fluorescent rhodamine-123. Isolated PMNs (1×10^6^/mL) were pre-incubated in MSCs (bmMSCs or pgMSCs) supernatant or standard culture medium for 1 h. After washing with 1× PBS, PMNs were incubated in the presence or absence of *E. coli* (strain JM109) with MOI 25, 50, 100, and 200 in RPMI-1640 supplemented with 10% autologous serum for 15 minutes at 37°C. Then, DHR-123 (2.5µg/mL) was added for 15 minutes and afterwards incubated on ice for 15 min. Analysis was performed immediately by flow cytometric measurements.

### Statistical analysis

The Mann-Whitney U test was used to statistically evaluate the difference between the groups (Figure 1–3). A *p* value <.05 was considered as significant (Figure 1–3). ANOVA test was used to statistically evaluate the difference between more than two groups (Figure 5–7). A *p* value <.05 was considered as significant. Data are represented using mean ± SD in all cases. Calculations were performed using Sigma Plot software (Version 12). For gene expression values ≥ 2.0 were set as a significant increase of gene expression and values ≤ 0.5 were set as a significant decrease of gene expression after LPS stimulation.
