# Correction: Mesenchymal Stem Cells Augment the Anti-Bacterial Activity of Neutrophil Granulocytes

**DOI:** 10.1371/journal.pone.0114201

**Published:** 2014-11-21

**Authors:** The PLOS ONE Staff

There is an error in the last sentence of the Methodology/Principal Findings section of the Abstract. The sentence should be: “Both types of MSCs augmented anti-microbial functions of PMNs. LPS stimulation, particularly of bmMSCs, further augmented MSC-mediated activation of PMN.”
